# 126 An examination of changes in an academic year in the Post-primary whole-school physical activity program

**DOI:** 10.1093/eurpub/ckae114.039

**Published:** 2024-09-26

**Authors:** Kwok Ng, Fiona McHale, Elaine Murtagh, Catherine Woods

**Affiliations:** Physical Activity for Health Research Centre, University Of Limerick, Limerick, Ireland; Lithuanian Sports University, Kaunas, Lithuania; University of Turku, Rauma, Finland; Physical Activity for Health Research Centre, University Of Limerick, Limerick, Ireland; Physical Activity for Health Research Centre, University Of Limerick, Limerick, Ireland; Physical Activity for Health Research Centre, University Of Limerick, Limerick, Ireland

## Abstract

Whole-school physical activity (PA) programmes are widely recommended for PA promotion by the international society of PA for health. In Ireland, the Active School Flag (ASF) is such a programme and is supported by both Departments of Education and Health with the purpose of increasing PA opportunities for the whole school community, focusing mainly on students. The ASF programme is research informed acting at multi-levels (students, staff and management) with multi-components including peer-led activities, student voice, environmental changes and whole-school events throughout the year. The purpose of this study was to compare outcomes between intervention and control schools after one year.

Students, from a random class from six schools, that was comprised of three intervention (n = 346) and three matched case control (n = 275), were recruited at baseline. Survey measures included PA behaviours and its correlates, such as screen time, perceived health, self-efficacy, intention for PA, peer support, scholastic competencies, student autonomy, and the health, mood and family subdomains of Kidscreen. The survey was administered at the beginning and end of the academic year. Mean differences between the two-time points within groups were tested through paired samples t-tests and between groups with repeated ANOVA.

**Findings:**

The overall characteristics between the two conditions were similar at baseline. A 72% retention rate (n = 442) made up the final dataset. At the end of the year, there was a statistically significant (p<.05) reduction in PA, self-efficacy, student autonomy, and family well-being among students in the intervention schools. Statistically significant (p<.05) reductions in expectations, peer support, student autonomy and mood among students in control schools (n = 173). There was a between group effect in perceive health (p<.001), whereby students in the intervention group increased levels of perceived health, but not among the students in control schools.

**Interpretations:**

This whole-school PA intervention is a complex study. According to the results, the programme shows evidence of promise on perceive health. These results from a pilot study pave way for conducting a larger study at scale.

